# Histone modification in *Saccharomyces cerevisiae*: A review of the current status

**DOI:** 10.1016/j.csbj.2023.02.037

**Published:** 2023-02-24

**Authors:** Kwon Young Chou, Jun-Yeong Lee, Kee-Beom Kim, Eunjeong Kim, Hyun-Shik Lee, Hong-Yeoul Ryu

**Affiliations:** a School of Life Sciences, College of National Sciences, Kyungpook National University, Daegu 41566, Republic of Korea; b BK21 Plus KNU Creative BioResearch Group, School of Life Sciences, College of National Sciences, Kyungpook National University, Daegu 41566, Republic of Korea

**Keywords:** PTM, post-translational modification, HAT, histone acetyltransferase, HDAC, histone deacetylase, AdoMet, S-adenosylmethionine, R Arg, arginine, JMJC, Jumonji C, S, serine, T, threonine, Y, tyrosine, SAGA, Spt-Ada-Gcn5 acetyltransferase, CTD, C-terminal domain, SUMO, small ubiquitin-like modifier, DSB, double-strand break, CAF-1, chromatin assembly factor-1, STUbL, SUMO-targeted ubiquitin ligase, E Glu, glutamic acid, K Lys, lysine, Histone acetylation, Histone methylation, Histone phosphorylation, Histone ubiquitylation, Histone sumoylation, Histone acylation

## Abstract

The budding yeast *Saccharomyces cerevisiae* is a well-characterized and popular model system for investigating histone modifications and the inheritance of chromatin states. The data obtained from this model organism have provided essential and critical information for understanding the complexity of epigenetic interactions and regulation in eukaryotes. Recent advances in biotechnology have facilitated the detection and quantitation of protein post-translational modification (PTM), including acetylation, methylation, phosphorylation, ubiquitylation, sumoylation, and acylation, and led to the identification of several novel modification sites in histones. Determining the cellular function of these new histone markers is essential for understanding epigenetic mechanisms and their impact on various biological processes. In this review, we describe recent advances and current views on histone modifications and their effects on chromatin dynamics in *S. cerevisiae*.

## 1 Introduction

In eukaryotes, chromosomal DNA is packaged into canonical nucleosomes composed of 145–147 bp DNA fragments wrapped around an octamer of histone proteins (two molecules each of histones H2A, H2B, H3, and H4), and the nucleosomes containing histone variants form a higher-order chromatin structure [1], [2]. Each histone contains a globular domain and structurally separated N- and C-terminal tails, which extend from the nucleosomal core [3]. These histone regions are subject to multiple post-translational modifications (PTMs), which are predominantly distributed in flexible histone tails [4]. As PTMs alter the properties of the amino acid residues in the histone substrates or serve as binding sites for certain chromatin factors, all aspects of chromatin function, including chromatin condensation, DNA replication, transcription, RNA splicing, and DNA repair, can be affected by histone modifications [5]. Dysregulation of histone modification is correlated with the pathogenesis and pathophysiology of various diseases; thus, several research groups have investigated its modulation to explore its potential as therapeutic and drug targets for disease treatment [6], [7].

**Fig. 1:**
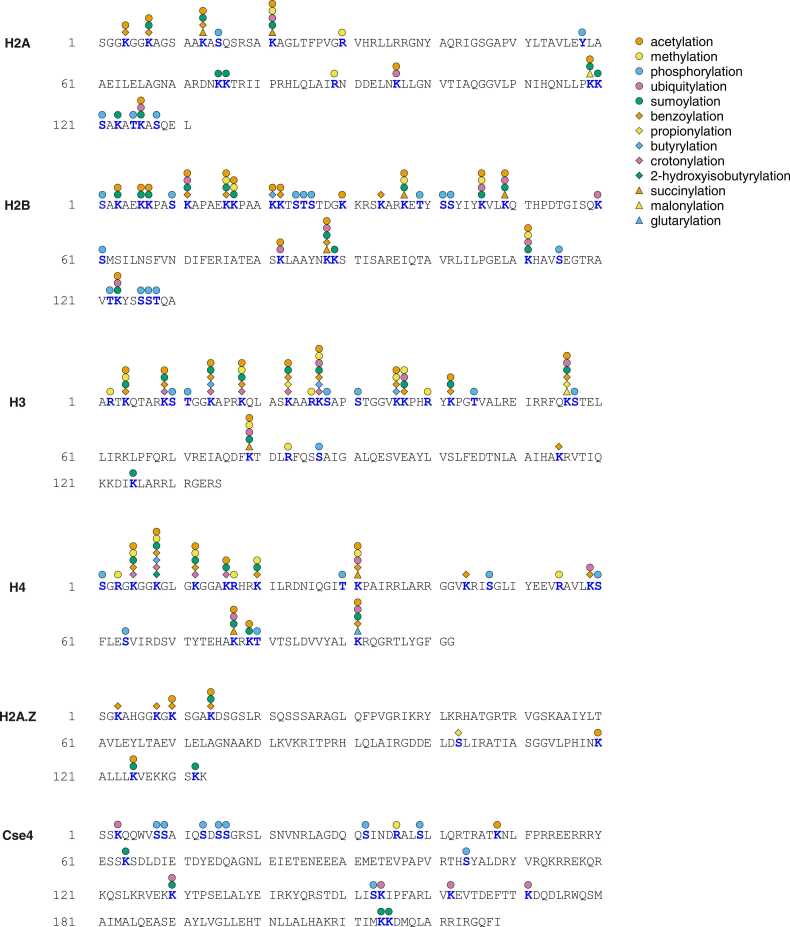
Representative diagram showing the distribution of post-translational modifications within histone core proteins and variants, H2A.Z and Cse4, in *Saccharomyces cerevisiae*. The types and specific sites of modifications are indicated.

The amino acid sequences of histones and their modifications are highly conserved in eukaryotes, from yeast to humans. Therefore, studies on yeast have provided valuable information on the mechanisms underlying epigenetic regulation and inheritance of chromatin states [8]. Notably, *Saccharomyces cerevisiae* is a well-characterized model system for studying histone modifications in chromatin dynamics [9]. Recent advances in biochemical and mass spectrometry technologies have led to the identification of various types of PTMs, including acetylation, methylation, phosphorylation, ubiquitylation, sumoylation, and acylation, at much more diverse sites in histones than was previously known [10] (Fig. 1 and Supplementary data 1). Accumulating evidence describing novel histone modifications and their modification sites provide novel insights into epigenetic research. Therefore, the aim of this review was to provide a current update on yeast histone modifications to better understand their cellular functions and relationships with various disorders.

## 2 Histone acetylation

Acetylation was the first discovered modification among various histone modifications and is the most widely studied in the field of epigenetic research [11]. Diverse lysine (Lys, K) residues on all histone subunits are known histone acetylation sites. Acetylation removes the positive charge of Lys on histones, thereby decreasing the interaction between histones and the negatively-charged phosphate groups of DNA, thereby leading to chromatin unfolding and gene activation [12]. In addition, acetylation of specific histone Lys residues provides binding sites for factors that interact with chromatin. Several transcription factors possess modification-interacting motifs, such as bromodomains, which bind to acetylated Lys residues [13].

Dynamic histone acetylation is modulated by the balance between histone acetyltransferase (HAT)-mediated transfer of acetyl groups from acetyl-CoA to the ε-amino group of Lys residues within histones and the removal of these acetyl groups by histone deacetylase (HDAC). Control of these enzyme activities influences the chromatin state and its functions [14], [15], [16]. Notably, histone acetylation preferentially occurs at promoter regions or 5′-transcribed genes and is highly correlated with the transcriptional activation as well as various pathways, including early firing of DNA replication, NuA4 histone acetyltransferase complex promoting DNA double-strand break repair, establishment and limitation of heterochromatin, H4K16 acetylation-dependent replicative lifespan control, H3K56 acetylation-controlled binding of histone chaperones Asf1 and Rtt106, DNA damage response, and interaction between the H3 variant Cse4 and inner kinetochore proteins Okp1 and Ame1 [17], [18], [19], [20], [21], [22], [23], [24]. Although the H2A variant H2A.Z is primarily located at the promoter regions of inactive genes, acetylation of its K14 residue is enriched in the case of certain active genes, suggesting a role of H2A.Z acetylation in its deposition during transcription [25].

## 3 Histone methylation

Specific methyltransferases transfer methyl groups from S-adenosylmethionine (AdoMet) to the ε-amino group of various Lys and arginine (Arg, R) residues on histone octamers and Cse4. This activity generates docking sites for various chromatin regulation factors, depending on which Lys/Arg residue is methylated [26]. Lysine methyltransferases can add up to three methyl groups to the same Lys residue, producing mono-, di-, or tri-methylation patterns, whereas arginine methyltransferases catalyze the formation of mono-, symmetric di-, or asymmetric di-methylation of Arg [26].

The role of methylation at K4, K36, and K79 on histone H3 in the transcription activation process has been well studied. These methylations are evolutionarily conserved in most eukaryotes [27]. H3K4 methylation is observed in a gradient across most active genes, with tri-methylation near the promoter, di-methylation at the 5′ end of the gene, and mono-methylation enriched further downstream, which is determined by the length of time the Set1/COMPASS methyltransferase complex spends near the nucleosome [28]. These different methylation patterns within genes distinctly affect their transcription, and H3K4 tri-methylation facilitates the recruitment of transcription activators. In contrast, H3K4 di-methylation provides a binding site for the Set3 HDAC complex, acting as a meiosis-specific repressor of sporulation genes and a suppressor of cryptic internal initiation within gene bodies [29], [30]. Although H3K4 mono-methylation represses transcription and is enriched at enhancer and promoter regions, it inactivates genes during nutrient stress [31]. In addition, H3R2 asymmetric di-methylation abrogates the binding of the Spp1 subunit of Set1/COMPASS, thereby blocking H3K4 tri-methylation [32].

H3K36 methylation by Set2 occurs co-transcriptionally [33], [34], and the tri-methylated form is enriched at the 3′ end of the gene [35]. Similar to H3K4 methylation, H3K36 methylation is closely related to transcription activation [36]. However, it can also negatively affect the transcription of certain genes [37] and prevent cryptic initiation by providing a memory signal for the recruitment of the Rpd3S HDAC complex [38], [39], [40], [41], [42]. Dot1, an H3K79 methyltransferase, was first identified as a telomeric silencing factor [43]; H3K79 methylation regulates the establishment of telomeric heterochromatin by antagonizing the spread of the Sir silencing complex [44], [45]. In addition to participating in telomere silencing, H3K79 methylation is observed in diverse active genes and is involved in transcriptional activation and elongation, cell cycle progression, and DNA damage response [46]. Set5 mono-methylates H4K5, K8, and K12 residues and has a functional link with Set1 [47], [48], [49]. R36 methylation of Cse4 is essential for kinetochore integrity and chromosome segregation [24], [50]. Recently, the methylation of several novel histone Lys/Arg residues, such as those in H2BK34 [51], H3K37 and K38 [52], H4R3 [53], and H4K20 [54], has been reported by several research groups. However, their functions have not yet been determined.

In *S. cerevisiae*, histone Lys methylation is reversible and dynamically regulated by enzymes containing the Jumonji C (JMJC) domains, namely Jhd1, Jhd2, Rph1, Gis1, and Ecm5 [55]. Jhd1 exhibits demethylase activity toward H3K36 mono- and di-methylations *in vitro* and *in vivo* [56] and is involved in transcription elongation by regulating H3K36 methylation of genes [57]. Jhd2 exhibits demethylase activity toward all forms of H3K4 methylations *in vitro* and *in vivo* [58], [59] and modulates telomeric silencing, rDNA condensation, and transcription [59], [60], [61]. Rph1 and Gis1 demethylases exhibit demethylase activity toward H3K36 tri- and di-methylation and H3K36 di- and mono-methylation *in vivo*, respectively [62]. Although the role of Rph1 is similar to that of Jhd1 in transcription [57], the function of Gis1 remains unclear [62]. Mammals secrete enzymes that convert methylated Arg residues into citrulline [63]. However, the regulatory mechanisms behind histone Arg demethylation in all eukaryotes have not been elucidated.

## 4 Histone phosphorylation

Histone proteins contain various evolutionarily conserved serine (S), threonine (T), and tyrosine (Y) residues that can be phosphorylated and dephosphorylated by kinases and phosphatases, respectively [64]. Histone phosphorylation is involved in a diverse range of nuclear events. Notably, its roles in DNA damage response and cell cycle regulation have been well established. Despite the identification of several phosphorylation sites in histone octamers, their biological function at each site remains an active area of investigation.

Shugosin functions as a centromeric protector of cohesion [65], resulting in the correct partitioning of chromosomes during cell division [66]. Bub1 kinase, which is required for proper chromosome segregation, catalyzes H2AS121 phosphorylation, leading to the proper localization of the shugosin protein. After a successful DNA repair process, the temporary cell cycle arrest in response to DNA damage is abrogated and switched to cell cycle resumption by Cla4 kinase-dependent H4T80 phosphorylation, co consequently promoting the recruitment of Rtt107, a competitor of checkpoint adaptor protein Rad9 [67], [68]. S phase-regulating kinase Cdc7-mediated H3T45 phosphorylation peaks during DNA replication, and its loss causes defects in DNA replication [69]. During yeast sporulation involving DNA compaction, H4S1 phosphorylation persists distinctly longer than H3S10 phosphorylation, which is required for meiotic chromosome condensation. H4S1 phosphorylation-dependent chromatin compaction is evolutionarily conserved in the later stages of spermatogenesis in *D. melanogaster* and mouse [70], [71]. Phosphorylation of H2AY58 and H3S57 regulates transcriptional elongation *via* coordination with the Spt-Ada-Gcn5 acetyltransferase (SAGA) complex and H3K56 acetylation, respectively [72], [73]. Several kinases can catalyze H3T11 phosphorylation to participate in various cellular processes, including Sch9 and CK2-mediated regulation of chronological lifespan [74]; Pyk1-driven association of the Sir complex with telomeric regions and prevention of Sir2 HDAC degradation by autophagy [75]; and Mek1, a regulator of meiotic recombination and progression upon DNA double-strand breaks, with unknown function [76]. Various serine residues of Cse4 are also phosphorylation targets, which are mainly catalyzed by Cdc5 for chromosome segregation [77], [78].

## 5 Histone ubiquitylation

Conjugation of the small protein ubiquitin to Lys side chains within target proteins is a prerequisite for proteasome-mediated protein degradation pathways. However, ubiquitin transfer to substrates, such as histones, also participates in signal transduction that regulates diverse biological processes [79]. Although global PTM analysis predicts several ubiquitylation sites on histones [80], the function of H2BK123 mono-ubiquitylation has only been determined in *S. cerevisiae*. Cooperation between the Rad6 E2-conjugating enzyme and the Bre1 E3 ligase leads to the conjugation of ubiquitin (activated by the E1 activation enzyme) to H2BK123. In contrast, this modification is dynamically regulated by two deubiquitinases, Ubp8 and Ubp10, which are involved in transcription activation and silencing mechanisms, respectively [81].

H2BK123 ubiquitination is involved in various chromatin functions, including gene expression, DNA replication, and DNA damage checkpoint response [81], [82], [83]. Conversely, H2BK123 ubiquitylation is required for optimal transcription activation in the transcription initiation stage, and Ubp8-mediated subsequent deubiquitylation facilitates transcription elongation. This is because persistent H2BK123 ubiquitylation impedes the association of Ctk1 RNA polymerase II C-terminal domain (CTD) kinase with active genes [84], [85], [86], [87]. Notably, H2BK123 ubiquitylation-dependent H3K4 and K79 methylation, an evolutionarily conserved trans-tail pathway, is the best-characterized histone crosstalk for maintaining dynamic chromatin structure during transcription [88], [89]. In addition, the association of Sir2 with telomeric regions is tightly regulated by the trans-tail pathway [90]. The loss of enzymes in the trans-tail pathway disrupts the silencing of the *URA3* reporter gene located at all heterochromatin loci [43], [90], [91], whereas both Ubp8 and Jhd2 H3K4 demethylases exhibit a specific anti-silencing function in the heterochromatic rDNA region [61], [90]. Furthermore, the trans-tail pathway-mediated formation of intact telomeric chromatin is required to maintain a normal cellular lifespan. The described histone modifications accumulate at the telomere-proximal regions in replicatively-aged cells, coincident with a reduction in the recruitment of Sir2 [90], [92]. In contrast, the role of H2BK123 ubiquitylation is independent from that of H3K4 and K79 methylation, as it enhances nucleosome stability during transcription elongation, which is consistent with the effect of H2B ubiquitination on nucleosome dynamics in humans [93], [94]. Moreover, Psh1 E3 ligase targets Cse4 to prevent its mislocalization [95].

## 6 Histone sumoylation

The covalent attachment of the small ubiquitin-like modifier (SUMO) protein to histone Lys residues is catalyzed by a three-enzyme cascade reaction involving Uba2/Aos1 E1, Ubc9 E2, and E3 ligases, similar to that in the ubiquitylation pathway [96]. Histone sumoylation plays crucial roles in various biological processes, such as cellular stress response, nucleocytoplasmic transport, and apoptosis, in addition to regulating chromatin function [97], [98], [99], [100], [101]. SUMO molecules can be assembled into polymers on histones, which are specifically disassembled by Ulp2 SUMO protease [102]. All four core histones, as well as the H2A.Z and Cse4, are substrates for sumoylation in *S. cerevisiae*, and multiple sumoylation sites have been identified [103], [104], [105], [106]. Known sumoylation sites of H2B are K6, K7, K16, and K17, whereas those of H4 are K5, K8, K12, K16, and K20; however, more sites are likely to exist within core histones [104].

Until recently, the role of histone sumoylation was assumed to be the repression of transcription by opposing gene activation markers, such as histone acetylation and ubiquitylation, or the recruitment of their enzymes [104], [107]. However, paradoxically, genome-wide histone sumoylation analysis has revealed that this modification is highly concentrated in several active genes, suggesting that histone sumoylation-mediated transcription control is complex [108]. The trans-tail pathway of H2B ubiquitylation and H3K4 methylation facilitates subsequent histone sumoylation during transcription [102], [108]. The presence of histone sumoylation also inhibits the recruitment of Ctk1 to chromatin, similar to the effect of H2B ubiquitylation on Ctk1 binding. Consequently, Ulp2-mediated histone desumoylation modulates the level of specific CTD phosphorylation of RNA polymerase II, which is required for the efficient transition between transcription initiation and elongation steps [102]. Consistent with the known pathway of H3K4 di-methylation-dependent control of the Set3 HDAC complex, histone sumoylation provides a binding platform for the Cpr1 subunit in the Set3 complex, resulting in a significant increase in the cryptic initiation of non-coding RNAs from internal sites within gene bodies [108]. Therefore, an elaborate histone modification network involving consecutive ubiquitylation, methylation, sumoylation, and deacetylation of histones is required to maintain transcriptional fidelity by suppressing spurious transcription [109].

Another possible role for histone sumoylation in chromatin dynamics was recently identified in *S. cerevisiae* [110]. The Remodeling the Structure of Chromatin (RSC) complex, an ATP-dependent nucleosome remodeler, regulates various nuclear pathways, including transcription, DNA replication, chromosome segregation, and DNA repair, by altering the location, occupancy, and composition of nucleosomes in chromatin [111], [112], [113], [114], [115], [116], [117], [118]. H3 K14 acetylation and H2B sumoylation are required for chromatin binding of RSC during chromosome segregation. However, the role of histone sumoylation in other RSC-controlled processes, such as transcription or replication, has not yet been determined [110].

Moreover, upon induction of persistent double-strand breaks (DSBs) in the absence of homologous DNA for repair, H2A.Z is rapidly loaded near the break site to drive the relocation of the unrepaired chromosomal ends toward the nuclear envelope and is gradually removed [105]. Although only a low level of SUMO-modified H2A.Z was originally reported in yeast cells during DSB repair [104], a subsequent study revealed that H2A.Z incorporated into nucleosomes at a persistent DSB site was sumoylated; this sumoylation was required for tethering of DSB to the nuclear periphery [105]. Although the mechanism underlying the effects of sumoylation of H2A.Z on DSB relocation has not been elucidated, a key recombination factor in the DSB response, namely Rad52, is a known SUMO substrate [119], [120].

Cse4 has been reported as a substrate for Siz1 and Siz2 SUMO ligases *in vitro* and *in vivo* [106]. Other biochemical studies have reported the major SUMO-Cse4 conjugation sites [121], [122]. Sumoylation at C-terminal K215/216 sites of Cse4 facilitates its association with the Cse4-specific histone chaperone Scm3 [122], thereby promoting Cse4 deposition at centromeres. The chromatin assembly factor-1 (CAF-1) complex also interacts with K215/216-sumoylated Cse4 and drives overexpressed Cse4 into incorrect, noncentromeric sites [122]. However, CAF-1 can also trigger the deposition of Cse4 into the centromeric region when *SCM3* expression is reduced [122]. Notably, sumoylation of Cse4 at a different site, namely K65, prevents the aberrant spread of Cse4 into euchromatin by providing a signal for ubiquitylation by the Slx5/Slx8 SUMO-targeted ubiquitin ligase (STUbL), thereby leading to the proteasomal degradation of Cse4.

## 7 Histone acylation

In recent years, besides histone acetylation, the addition of diverse acetyl moieties to several Lys residues of histones has been identified and demonstrated to be associated with the regulation of various biological events [123]. These new types of histone marks, including benzoylation, propionylation, butyrylation, crotonylation, succinylation, and malonylation, were identified in *S. cerevisiae*. Benzoylation, propionylation, butyrylation, and crotonylation are examples of hydrophobic acylation, which increase the hydrophobicity and steric hindrance potential of modified lysines. Conversely, acidic modifications, such as succinylation and malonylation, alter the charge on Lys from positive to negative [124].

The 27 Lys sites on core histones and four sites on H2A.Z are targets of benzoylation, and their levels are dynamically regulated by the SAGA complex and NAD^+^-dependent HDAC Hst2 [125]. Multiple Lys sites for propionylation and butyrylation of histones have also been identified by mass spectrometry [126]; however, contrary to studies in other eukaryotes, cellular and molecular functions of histone benzoylation, propionylation, and butyrylation have not yet been determined in *S. cerevisiae*. Gcn5 in SAGA and Esa1 in the NuA4 HAT complex can catalyze crotonylation at nine Lys residues on histones and promote the transcription of crotonylation-dependent genes [127]. The eight Lys residues within histone octamers have been proposed as succinylation sites [128], [129], [130]. Substitution of certain succinylated Lys sites in histones with glutamic acid (Glu, E) to mimic constitutively succinylated Lys in yeast cells leads to potential phenotypes related to nucleosome instability, such as a severe growth defect in H4K31E, sensitivity to the DNA-damaging agent in H2AK21E, and heat-sensitivity and loss of silencing at rDNA and telomere loci in H4K77E [128], [130]. H2AK119 malonylation inhibits the interaction between Bub1 kinase and H2A, thereby decreasing Bub1-dependent H2AS121 phosphorylation and chromosomal localization of shugoshin proteins required for proper chromosome segregation [131]. Although histone β-hydroxybutyrylation was first observed in 2016, the modification sites and their associated functions have not yet been comprehensively elucidated [132]. However, the addition of 2-hydroxyisobutyryl, an isomer of β-hydroxybutyryl, to H4K8 was previously reported [133].

## 8 Summary and outlook

DNA methylation and histone modifications are well-explored epigenetic mechanisms for stable alterations in gene expression without any underlying modifications in the actual genetic sequence [134]. *S. cerevisiae* is considered a valuable eukaryotic model for studying the role of histone modification in chromatin structure and function despite the lack of DNA methylation/demethylation machinery [135]. Moreover, the study on histone modifications in *S. cerevisiae* provides information about their function and regulation [136] because histone modification patterns, such as enrichment of H3K4 and other histone acetylation, on active genes are well-conserved in various organisms, including yeast, *D. melanogaster*, and humans. Biotechnological advances in the detection and quantitation of protein modifications have led to the discovery of various novel histone modifications and their positions in all eukaryotes, including *S. cerevisiae*. However, several novel histone markers require further validation. For example, although histones covalently modified by ethylation, thiophosphorylation, piperidination, or 2-amino-3-oxobutanoic acid have been reported in *S. cerevisiae* [126], additional empirical investigation is required to validate these modifications. Although histone Arg methylation and its catalytic enzymes have been reported in *S. cerevisiae*, there is no clear yeast homolog of histone deaminase PADI4, which metabolizes unmethylated Arg to citrulline [137]. Therefore, an approach to identifying a potential demethylase is required to establish the field of yeast histone deamination [126]. Each histone modification is considered to act as an independent signaling molecule; however, recent research has confirmed that various modifications at different sites on histones can exhibit interdependence and inherent interaction patterns under specific conditions, which have crucial implications for the control of chromatin function [138]. Moreover, histone modifications and their crosstalk are essential for various cellular processes in all eukaryotes and are implicated in diverse pathogeneses and diseases, such as neurodegenerative diseases and cancer [7], [139], [140], [141], [142]. Hence, improved characterization and discovery of histone modifications will likely lead to the development of novel therapies and serve as attractive drug targets for disease treatment.

## CRediT authorship contribution statement

**Kwon Young Chou:** Conceptualization, Writing - review & editing. **Jun-Yeong Lee:** Writing – review & editing. **Kee-Beom Kim:** Writing – review & editing. **Eunjeong Kim:** Writing – review & editing. **Hyun-Shik Lee:** Writing – review & editing. **Hong-Yeoul Ryu:** Conceptualization, Funding acquisition, Writing - review & editing.
